# Structural and functional characterization of the divergent *Entamoeba* Src using Src inhibitor-1

**DOI:** 10.1186/s13071-017-2461-5

**Published:** 2017-10-18

**Authors:** Luilli López-Contreras, Verónica Ivonne Hernández-Ramírez, Mayra Herrera-Martínez, Sarita Montaño, Luis Alejandro Constantino-Jonapa, Bibiana Chávez-Munguía, Patricia Talamás-Rohana

**Affiliations:** 1 Área Académica de Medicina, Instituto de Ciencias de la Salud, Universidad Autónoma del Estado de Hidalgo, Camino a Tilcuatla s/n Municipio de San Agustín Tlaxiaca. C.P, 42160 Pachuca de Soto, Hidalgo Mexico; 2 Departamento de Infectómica y Patogénesis Molecular, Centro de Investigación y de Estudios Avanzados del I.P.N, Avenida Instituto Politécnico Nacional No. 2508, Col. San Pedro Zacatenco, Delegación Gustavo A. Madero, 07360 CDMX, CP Mexico; 3 Facultad de Ciencias Químico Biológicas de la Universidad Autónoma de Sinaloa, Calz. de las Américas Norte 2771, Burócrata, 80030 Culiacán de Rosales, Sinaloa Mexico

**Keywords:** Actin cytoskeleton, *Eh*Src, *Entamoeba* spp., Src inhibitor-1

## Abstract

**Background:**

The abundant number of kinases that *Entamoeba histolytica* possesses allows us to assume that the regulation of cellular functions by phosphorylation-dephosphorylation processes is very important. However, the kinases responsible for the phosphorylation in *Entamoeba* spp. vary in the structure of their domains and, therefore, could be responsible for the unusual biological characteristics of this parasite. In higher eukaryotes, Src kinases share conserved structural domains and are very important in the regulation of the actin cytoskeleton. In both *Entamoeba histolytica* and *Entamoeba invadens*, the major Src kinase homologue of higher eukaryotes lacks SH3 and SH2 domains, but does have KELCH domains; the latter are part of actin cross-linking proteins in higher eukaryotic cells.

**Methods:**

The function of the *Eh*Src protein kinase of *Entamoeba* spp. was evaluated using Src inhibitor-1, microscopy assays, Src kinase activity and western blot. In addition, to define the potential inhibitory mechanism of Src-inhibitor-1 for the amoebic *Eh*Src protein kinase, molecular dynamic simulations using NAnoscale Molecular Dynamics (NAMD2) program and docking studies were performed with MOE software.

**Results:**

We demonstrate that Src inhibitor-1 is able to prevent the activity of *Eh*Src protein kinase, most likely by binding to the catalytic domain, which affects cell morphology via the disruption of actin cytoskeleton remodeling and the formation of phagocytic structures without an effect on cell adhesion. Furthermore, in *E. invadens*, Src inhibitor-1 inhibited the encystment process by blocking RhoA GTPase activity, a small GTPase protein of Rho family.

**Conclusions:**

Even though the *Eh*Src molecule of *Entamoeba* is not a typical Src, because its divergent amino acid sequence, it is a critical factor in the biology of this parasite via the regulation of actin cytoskeleton remodeling via RhoA GTPase activation. Based on this, we conclude that *Eh*Src could become a target molecule for the future design of drugs that can prevent the transmission of the disease.

**Electronic supplementary material:**

The online version of this article (10.1186/s13071-017-2461-5) contains supplementary material, which is available to authorized users.

## Background

In humans, *E. histolytica* causes invasive amoebiasis, which is an important source of morbidity and mortality in developing countries [1, 2]. *Entamoeba invadens* is the most important protozoan pathogen of reptiles [3].

*Entamoeba* spp. are simple eukaryotes that lack classical defined mitochondria, rough endoplasmic reticulum or Golgi bodies. However, trophozoites have very elaborate signaling machinery, through which they “feel” and interact with the different environments they find as they progress during invasion. These signaling pathways help the parasite to respond adequately to favor invasion and, therefore, are generally considered to be an important part of the interaction that the parasite establishes with the host [4, 5].

One of the most studied species in the genus *Entamoeba* is *E. histolytica*. In this protozoan parasite, the family of kinases is very abundant and these are part of the signaling systems of the parasite. The abundant number of kinases that *Entamoeba histolytica* possesses allows us to assume that the regulation of cellular functions by phosphorylation-dephosphorylation is very important. However, the kinases responsible for the phosphorylation processes in *Entamoeba* vary in the structure of their domains and, therefore, could be responsible for the unusual biological characteristics of this parasite [5, 6].

An example of these proteins is a homologue of the Src tyrosine kinase. Src kinases from higher eukaryotes share a conserved domain structure that consists of the consecutive peptide binding domains SH3, SH2, tyrosine kinase (SH1) domains, and SH4 domain; the last one always myristoylated, thus allowing the enzyme to be associated with the cell membrane. In the main homologue of the Src kinase from *E. histolytica*, SH3 and SH2 domains are absent. However, this molecule possesses KELCH domains, which strongly suggest its involvement in cytoskeleton regulation [6, 7]. These characteristics of the *Eh*Src protein kinase indicate that this enzyme diverges significantly from the closest homologous present in higher eukaryotes. Nevertheless, through the use of Src inhibitor-1, previous work from our group demonstrated its importance in amoebic movement and phagocytosis, as well as its role in the parasite’s virulence [8].

Src inhibitor-1 (4-(4′-phenoxyanilino)-6,7-Dimethoxy-*N*-(4-phenoxyphenyl)-4-quinazolinamine) is a potent, selective and competitive dual site (both ATP and peptide binding) Src kinase inhibitor that has been used to define physiological roles for Src family members in mammalian cells [9].

In this work, we describe structural and functional characteristics of amoebic Src. We demonstrate that Src inhibitor-1 diminishes Src activation in *E. histolytica*, which affects actin cytoskeleton remodeling, especially stress fiber formation and phagocytosis via defects in the formation of the phagocytic invaginations, without an effect on amoebic adhesion. Moreover, during the encystment in vitro of the model parasite *E. invadens*, the blocking of Src activity by Src inhibitor-1 affected encystment through the inhibition of RhoA activation. Therefore, this peculiar molecule and the significance of *Eh*Src or *Ei*Src activity in the biology of the parasite raise questions regarding its regulation and the potential to use *Eh*Src protein as a drug-target to prevent *Entamoeba* transmission.

## Methods

### Phylogenetic analysis

Phylogenetic and alignment analyses were conducted in the ClustalW package [10] with the amino acid sequences of 10 Src proteins from different species (*E. histolytica*, *E. invadens*, *C. elegans*, *Mus musculus* and *Homo sapiens*) obtained from the NCBI GenBank (http://www.ncbi.nlm.nih.gov/). The sequence alignment and Neighbor-Joining tree were then manually edited [11, 12].

### 3D structure generation

The 3D structure of the *Eh*Src protein was built using I-TASSER server [13, 14], which yielded a cluster of different models from which the most energetically stable was selected. The following crystal structures were used as templates: *Homo sapiens* tyrosine protein kinase ZAP-70 (GenBank: 2OZO), *Homo sapiens* proto-oncogene tyrosine-protein kinase ABL1 (GenBank: 2FO0), *Homo sapiens* Tyrosine-protein kinase SYK (GenBank: 4FL2), *Homo sapiens* Tyrosine-protein kinase SRC (GenBank: 1FMK), *Homo sapiens* fibroblast growth factor receptor 2 (kinase domain) (GenBank: 2PSQ), *Mus musculus* proto-oncogene tyrosine-protein kinase ABL1 and the SH3-SH2 kinase domain (GenBank: 1OPK). The error associated with creating models using sequences and ab initio was refined by 100 ns of MD simulations to avoid bad internal atomic contacts. The structural alignment of the kinase domain between *Eh*Src and 2SRC (*Homo sapiens*) was performed in the Visual Molecular Dynamics (VMD) program [15, 16]. The ligand ISRC was built with Gaussian 03 using AM1 base [17].

### Molecular dynamics simulations

The molecular dynamics (MD) simulations were performed in the Hybrid Cluster Supercomputer “Xiuhcoatl” at CGSTIC-CINVESTAV (http://clusterhibrido.cinvestav.mx/), using NAMD 2.8 [18], which employed the CHARMM27 [19] using periodic boundary conditions in the MD simulations; Particle Mesh Ewald was used to account for electrostatic interactions [20]. The hydrogen atoms were added using the software psfgen from the VMD program [21]. The system was subjected to energy of minimization for 1000 steps followed by equilibration for 1 ns; the simulations were subsequently continued without restraints. The long MD simulations were run for 100 ns using the canonical NTV ensemble [22].

### Trajectory analysis

The Carma program [23] was used for structural analysis to obtain the root mean square deviation (RMSD), the root mean square fluctuation (RMSF) and the radius of gyration (Rg), as well as the snapshot (100 ns) used for docking purposes [24].

### Docking analysis

The protein-ligand docking studies, molecular graphics and protein visualizations were performed using the Molecular Operating Environment (MOE) program [25]. The studies for protein-protein docking were performed using Cluspro server [21]. Molecular graphics work and protein visualizations were performed with VMD.

### *Eh*Src antiserum production

For the generation of a monospecific polyclonal antibody against *Eh*Src, the methodology described [26] was generally followed. A peptide (KEEISEDDGYGETQEE) from the catalytic domain of *Eh*Src (GenBank: EAL46348.1) was designed. The immunograde peptide was purchased from GL BIOCHEM, Shanghai Ltd. and used for rabbit immunization. A female white New Zealand rabbit was immunized 4 times with 1 week intervals for each injection. In the first immunization, 1 mg peptide and 500 μl of Freund’s complete adjuvant (Sigma-Aldrich, San Luis, MO, USA) were mixed and injected subcutaneously. For the subsequent immunizations, 1 mg peptide was mixed with Freund’s incomplete adjuvant (Sigma-Aldrich, San Luis, MO, USA) and injected. Before each immunization, blood was drawn by venous puncture from the rabbit ear and allowed to clot for 2–3 h at room temperature before preparation of sera. Titration of the specific polyclonal antibody was performed using Western blot (data not shown).

### Parasite cultures and in vitro encystation

Trophozoites of *E. histolytica* (HM1-IMSS) and *E. invadens* (IP-1) were cultivated in TYI-S-33 medium [27] supplemented with 10% (*v*/*v*) bovine serum (BS) and 3% (*v*/*v*) Diamond vitamin-tween 80 solution (JRH Biosciences, Inc. St Louis, MO, USA) for 48 h in glass screw cap tubes (16 × 125 mm) at 37 °C. Trophozoites were subsequently incubated on ice for 10 min, collected by centrifugation at 1100 *rpm* for 10 min, and washed three times in TYI-S-33 medium without serum. Required number of trophozoites were then treated separately with 1% DMSO or Src inhibitor-1 (30 μM) (Merck, Darmstadt, Germany) for 2 h at 37 °C and washed with TYI-S-33 medium without serum.

To induce encystment, *E. invadens* trophozoites harvested in the logarithmic phase of growth (5 × 10^5^/ml) were transferred to low glucose encystation medium (TYI without glucose) diluted to 47% with 5% bovine serum as described previously [28]. Trophozoites with or without Src inhibitor-1 (30 μM) treatment were incubated at 26 °C and, after 96 h, cysts were pelleted by sedimentation. The pellet was then re-suspended in 1 ml of 0.2% *w*/*v* Triton X-100 for 5 min. Cell counts were performed using a hemocytometer, and the number of detergent-resistant cysts was determined. Cysts were fixed and stained with white Calcofluor m2r (Sigma-Aldrich, St. Louis MO, USA), a fluorescent dye with specific binding to chitin molecules.

### *Entamoeba histolytica* Adhesion assays

As described earlier by López-Contreras et al. [8], after being treated with Src inhibitor-1 or DMSO for 2 h, trophozoites (2 × 10^5^) were washed with TYI-S-33 without BS to eliminate drug residues, and then the cells were incubated in TYI-S-33 without BS for 10 min in 96-well black plates (BD Cellware, Bedford, MA, USA) at 37 °C. Non-adhered cells were eliminated, and adhered cells were stained with Sytox Green (1:5000) (Molecular Probes, Eugene, OR, USA), then adhered cells were washed with PBS and fluorescence was measured at 488 nm in a fluorometer (Fluoroskan Ascent FL, Thermo, Boston, MA, USA). Fluorescence from DMSO-treated trophozoites was defined as 100% of adhesion.

### Immunoprecipitation and western blot assays

Immunoprecipitation assays were performed as previously described [29]. Briefly, trophozoite lysates (1 mg of total protein) were precleared with protein G-agarose (Gibco-BRL, Grand Island, NY, USA) (previously blocked with 2% bovine serum albumin) for 2 h at 4 °C. The anti-*Eh*Src serum (1/1000) was then added to the cell lysates supernatant. Mixtures were incubated overnight at 4 °C, and then 2% bovine serum albumin (BSA) blocked protein G-agarose was added and incubated for another 2 h at 4 °C. Agarose beads were recovered by centrifugation at 11,600× *g* for 2 min at 4 °C, washed with 10 mM Tris-HCl pH 7.4, containing 150 mM NaCl, 3 mM EDTA, and 1% Nonidet P-40, resuspended in Laemmli’s sample buffer, and boiled for 5 min. After centrifugation, supernatants were loaded onto a 10% SDS-polyacrylamide gel electrophoresis and then processed as described previously with anti-phosphotyrosine (1/1000), anti-actin (1/1000), anti-*Eh*Src (1:50,000) antibodies and their respective secondary antibodies.

### Src kinase activity assays

After immunoprecipitation (1 mg of total protein) with *Eh*Src antibody (1:1000), immunoprecipitates were analyzed using a Pro-Fluor® Src-Family kinase assays (Promega, Fitchburg, WI, USA) according to manufacturer’s instructions. The fluorescence was read at a wavelength of 525 nm and Src kinase activity present in immunoprecipitates of DMSO-treated trophozoites was defined as 100%.

### Confocal microscopy assays

Analyses by confocal microscopy were done as described previously [30], with slight modifications. Trophozoites incubated on coverslips, with or without Src inhibitor-1 treatment, were fixed with 4% formaldehyde, blocked with BSA for 1 h at 37 °C, and incubated overnight with an anti-phospho-Src antibody (1/50) (Cell Signaling Technology, Danvers, MA, USA) or anti-*Eh*Src (1/250). Cells were then washed with PBS and incubated with Fluorescein-isotiocyanate (FITC)-labeled goat anti-rabbit immunoglobulin G (IgG) (1/100) (Jackson ImmunoResearch, Pennsylvania, USA) secondary antibody. Actin was stained with rhodamine-phalloidin (1:25) (Molecular Probes, Oregon, USA) for 30 min at 37 °C. Coverslips, which were mounted with Vectashield (Vector Laboratories, Ontario, Canada), were analyzed by confocal microscopy in a Laser Scan Microscope 700 (Carl Zeiss, Oberkochen, Germany). The intensities of fluorescence were obtained with the software ZEN BLUE 2017 and the Pearson’s coefficient was calculated with the SPSS version 24.

### Scanning and transmission electron microscopy assays

As described by González-Robles et al. [31], trophozoites with or without Src inhibitor-1 treatment were fixed with 2.5% glutaraldehyde in 0.1 M sodium cacodylate buffer, pH 7.2, dehydrated with increasing concentrations of ethanol, and critical point dried using a Samdri apparatus. Then, samples were gold coated in an ion-sputtering device (JOEL-JFC-1100, Jeol USA Inc., Peabody, MA, USA) and examined with a Zeiss DSM 982 Gemini scanning electron microscopy. For transmission electron microscopy, samples were fixed in 2.5% (*v*/*v*) sodium cacodylate buffer, pH 7.2, for 1 h, and then they were post-fixed for 1 h with osmium tetroxide in the same buffer. After dehydration in increasing concentrations of ethanol and propylene oxide, samples were embedded in polybed epoxy resins and polymerized at 60 °C for 24 h. Thin sections (i.e. 60 nm) were contrasted with uranyl acetate and lead citrate prior to examination in a Joel JEM-1011 transmission electron microscope.

### Measurement of RhoA activity

RhoA activity was determined using a luminescence based G-LISA™ RhoA activation assay kit (Kit #BK121, Cytoskeleton, Inc., Denver, CO, USA), according to the manufacturer’s instructions. This assay employs a GTP-binding protein that coats the wells of a 96-well plate. Active GTP-bound GTPases in cell lysates bind to the wells, whereas inactive GDP-bound GTPases are removed through washing steps. The bound active protein is subsequently detected by incubation with a specific antibody followed by a HRP-conjugated secondary antibody and a detection reagent, after which the luminescence is read on a microplate luminescence reader. A calibration curve with a positive control was performed, and the results are shown as ρg of protein-GTP/μg protein extract.

### Statistical analysis

Data are representative of two independent experiments, each one done in triplicate. The significant differences of experimental compared with control samples were analyzed by Student’s *t*-test using the Statistical Package for the Social Sciences (SPSS), version 24.

## Results and discussion

### Homology modeling of the divergent *Eh*Src

**Fig. 1:**
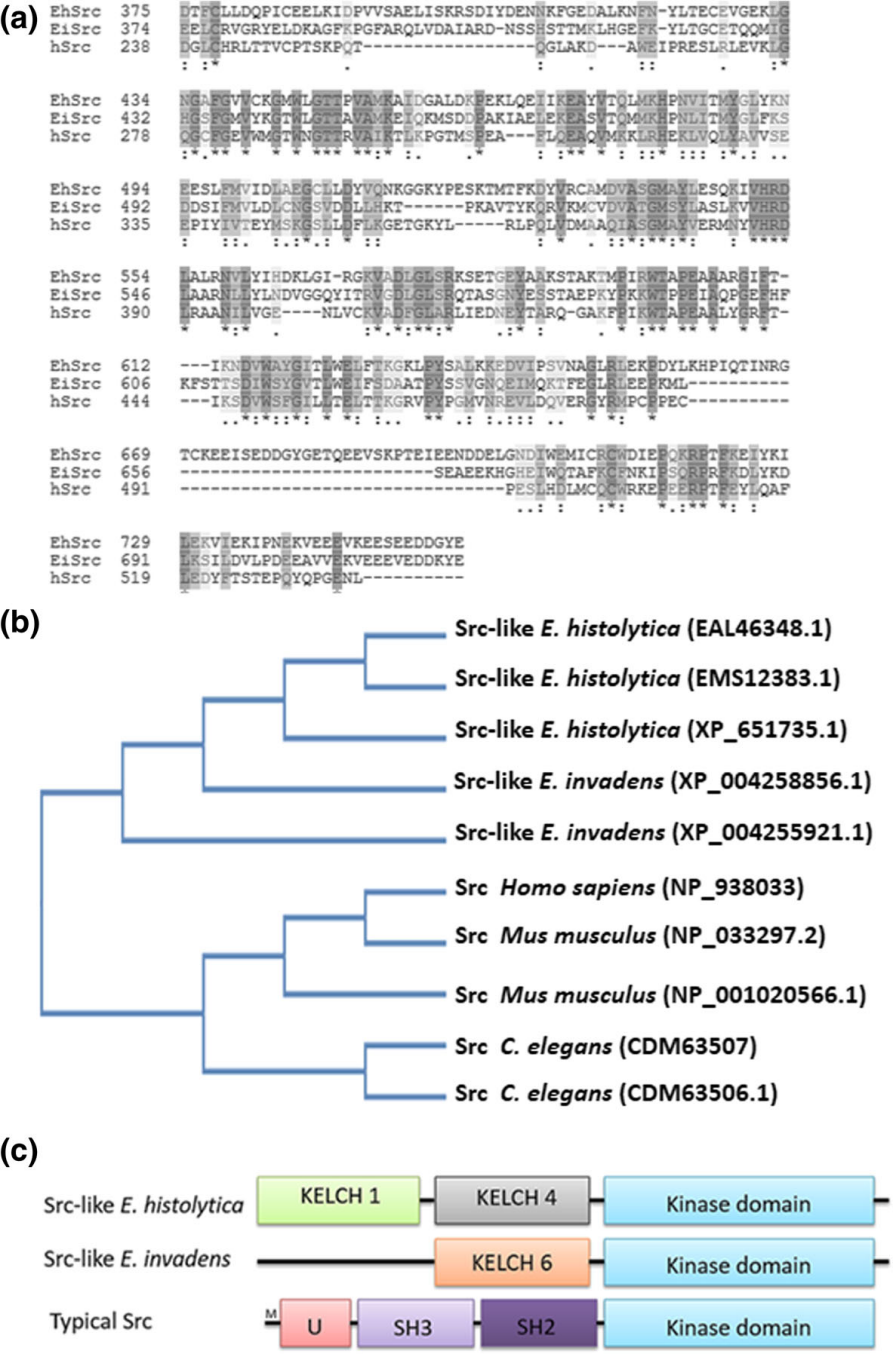
*Eh*Src is highly divergent from its closest homologues. **a** Alignment of the conserved domain among *Eh*Src (EAL46348.1), *Ei*Src (XP_004258856.1) and human Src (NP_938033). Identical amino acid residues are in dark gray and similar amino acid residues in light gray. **b** Phylogenetic tree of Src proteins of *E. histolytica*, *E. invadens*, *Homo sapiens*, *Mus musculus* and *C. elegans*. *Eh*Src protein of *E. histolytica* shows the highest homology with the Src protein of *E. invadens*, and these two proteins are poorly related with Src proteins from mammals as well as the nematode *C. elegans*. **c** Structural domains of typical Src and *Eh*Src proteins from *E. histolytica* and *E. invadens* are shown. The typical Src is composed of an amino-terminal myristoylation sequence (M), a unique region (U), Src-homology-2 (SH2) and SH3 protein-interaction domains; the Src of *Entamoeba* lacks these domains with the exception of the kinase domain

A BLAST search of human Src (hSrc) (GenBank: NP_938033) in the genomes of *E. histolytica* and *E. invadens* led to the identification of several kinases. The alignment between *E. histolytica* Src (*Eh*Src; GenBank: EAL46348.1) and *E. invadens* Src (*Ei*Src; GenBank: XP_004258856.1) showed a 29.135% homology between them. In comparison, the alignment between hSrc and *Eh*Src, presented a 15.013% homology, whereas the alignment between hSrc and *Ei*Src, showed a 13.87% homology (Fig. 1a). When performing a phylogenetic analysis it was evident that *Eh*Src and *Ei*Src are highly divergent from their closest homologues in higher eukaryotes (Fig. 1b). It has been described that the Src protein of *E. histolytica* has a kinase domain (Fig. 1c), lacks SH2 or SH3 domains but has KELCH 1 and KELCH 4 domains [6], whereas the Src protein from *E. invadens* (GenBank: XP_004258856.1) also lacks SH domains but has a KELCH 6 domain (Fig. 1c). KELCH proteins and KELCH-like proteins are a group of proteins that contain multiple KELCH motifs that form β-propellers to undergo a variety of binding interactions with other proteins, notably the actin filaments of a cell [32].

**Fig. 2:**
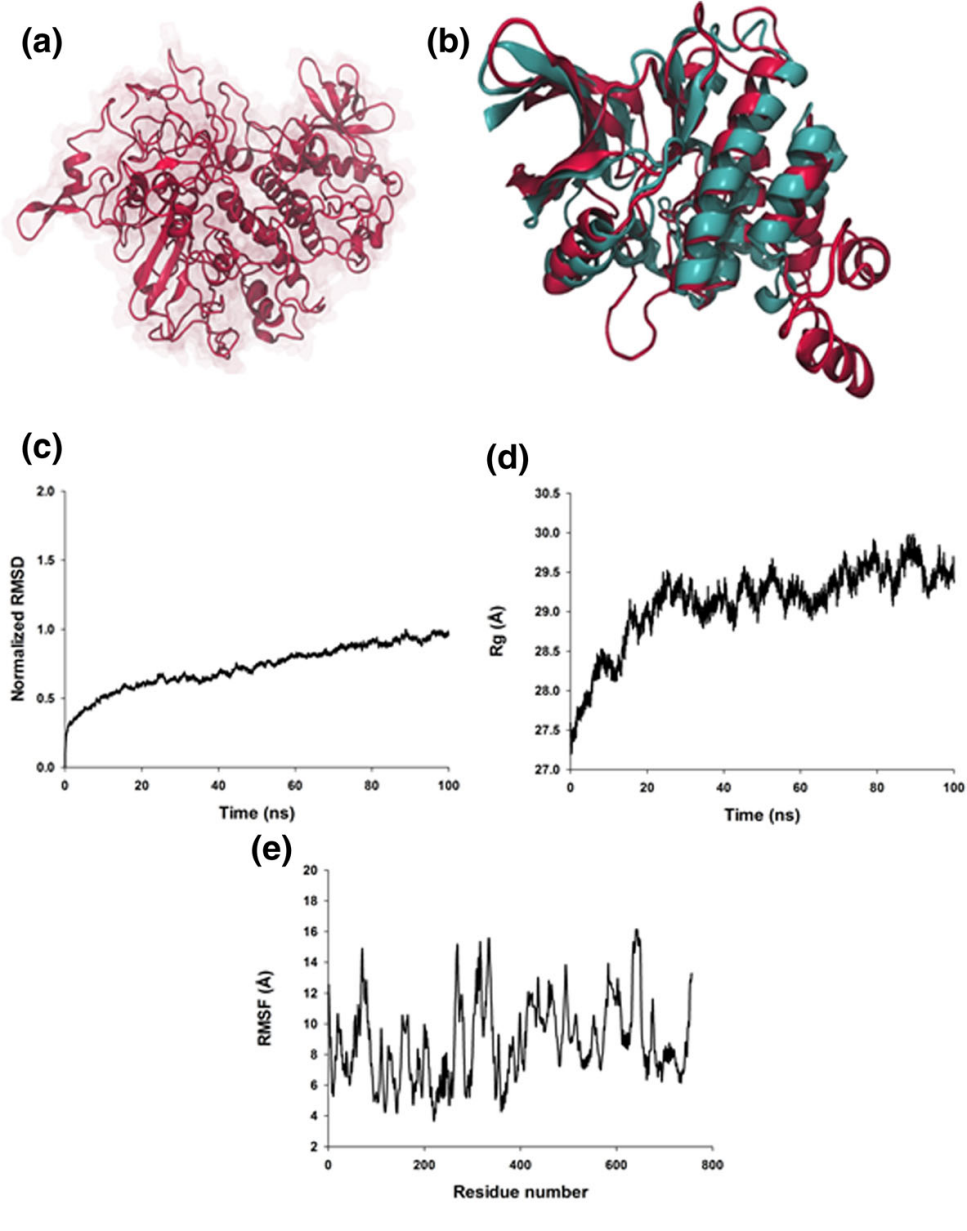
Homology structural modeling of *Eh*Src. **a** 3D model of *Eh*Src. **b** The structural alignment of *Eh*Src kinase domain in red and 2SRC kinase domain in cyan. **c** RMSD of *Eh*Src at 100 ns of trajectory on MD simulation. **d** Rg of *Eh*Src at 100 ns of trajectory on MD simulation. **e** RMSF of *Eh*Src at 100 ns of MD simulation

The 3D structure of *Eh*Src (GenBank: EAL46348.1) was performed using I-TASSER server [12]. It presented a normalized Z-score of 3.29 for 2OZO; 2.82 for 2FO0; 4.22 for 2SRC; 2.24 for 4FL2; 3.03 for 1FMK; 0.81 for 2PSQ; and 4.22 for 1OPK. Alignment was with a normalized Z-score > 1, meaning a good alignment. According to the 3D model obtained from the I-TASSER server, *Eh*Src protein is a globular protein that contains 750 amino acid residues (Fig. 2a). After 100 ns of MD simulations, the protein exhibits sixteen α-helices and thirteen β-sheets, whereas the rest of the structure is formed by coil structures. In addition, Tyr 586 (amino acid equivalent to Tyr 416 in a typical Src) within the catalytic site (constituted by the 430 to 730 amino acid domain) in the *Eh*Src protein, has the potential to be phosphorylated during activation.

**Fig. 3:**
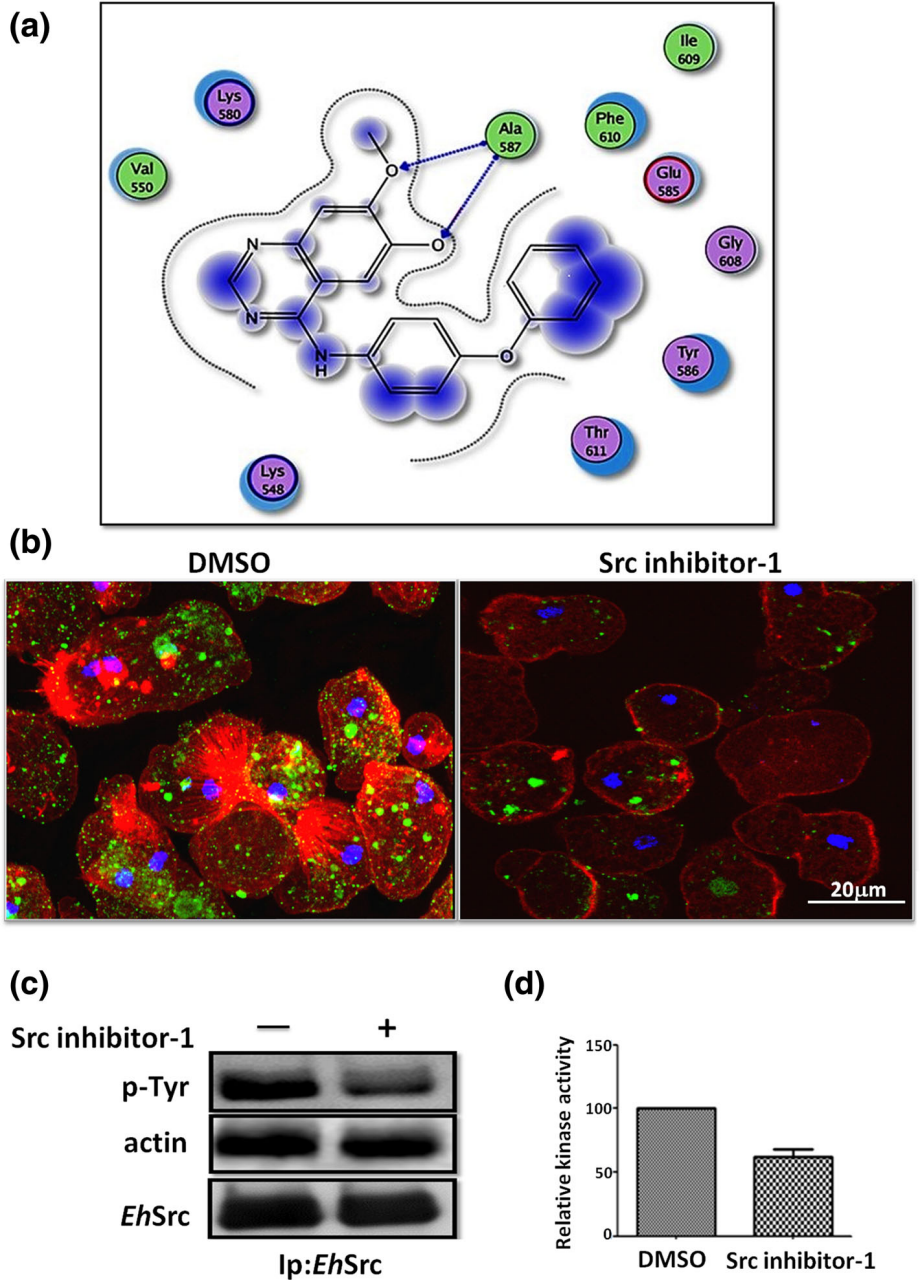
Src inhibitor-1 inhibits *Eh*Src protein activation of *E. histolytica* in silico and in vivo. **a** Predicted binding site of Src inhibitor-1 in the *Eh*Src protein of *E. histolytica*. **b** Confocal microscopy analysis of trophozoites with or without Src inhibitor-1 (30 μM) treatment. Actin was stained with rhodamine-phalloidin (red) (1/25), the nucleus was identified with DAPI 1/300 (blue) and p-Src (green) was detected with an anti-p-Src antibody (1/50). **c** Detection by western blot of phosphorylated tyrosine (p-Tyr), actin (α-actin) and *Eh*Src in *Eh*Src immunoprecipitated complex from treated and non-treated trophozoites with anti-phospho-tyrosine (1/1000), anti-actin (1/5000), and anti-*Eh*Src (1/5000) antibodies. **d** The kinase activity of DMSO- and Src inhibitor-1-treated trophozoites was determined using a Pro-Fluor® Src-Family kinase assays (Promega, Fitchburg, WI, USA) according to manufacturer’s instructions; the kinase activity from DMSO-treated trophozoites was defined as 100%. Values for each group represent mean ± SEM results from three independent experiments

The kinase domain (from 430 to 730 amino acids) of the 3D model obtained with I-TASSER for *EhSrc* and the crystal structure of human tyrosine kinase (2SRC) from 269 to 519 amino acids were structurally aligned (Fig. 2b) and the value of RMSD (distances between residues) was 0.765. This result suggests that the kinase domain is conserved in the *Eh*Src protein kinase; this region in the human tyrosine kinase contains an Y416 which is a phosphorylation site, whereas in the *Eh*Src protein this Y residue is in the 586 position. This would explain why pSrc antibody against human Src recognized the phosphorylated form of *Eh*Src (Fig. 3b).

To predict the place where Src inhibitor-1 is binding to the *Eh*Src molecule in amoebic trophozoites, MD simulations using NAMD2 were performed and the docking studies were done using Moe software.

The MD simulations results of *Eh*Src were submitted to structural analyses, such as the RMSD, the Rg and the RMSF as described [33]. The RMSD trajectory analysis shows the convergence during the MD simulation and its normalization reveals whether the values pursue a normal distribution. Normalized RMSD of Src indicates that the protein reached the equilibrium after 80 ns of MD simulation (Fig. 2c). Other *E. histolytica* proteins have been modeled and MD simulated just up to 10 ns [34, 35], this MD simulation analyses up to 100 ns are enough to predict reliable models for these proteins.

The Rg is a geometrical parameter that defines the protein expansion and compactness; we thus evaluated the compactness of the system. The Rg increased in the first 0 to 25 ns, and from 30 to 60 ns the slope was constant; however, from 60 to 70 ns, it increased, and from 70 to 100 ns, it became almost constant (Fig. 2d).

Additionally, we located the protein flexible regions via measurement of the RMSF values of the Cα backbone (Fig. 2e). The principal peaks of the flexible regions were located the first one, in 50 to 100 amino acid residues, with a displacement from 5 to 15 Å. The second peak was located at 250 to 350 amino acid residues, where the displacement changed from 5 to 15 Å. The third region was identified at 400 to 520 amino acids with a displacement of 7 to 13 Å. The last peak, where Tyr 586 is located, was identified from 560 to 650 residues, and it presented a displacement of 7 to 15 Å (Fig. 2e). These last regions belong to the catalytic domain, which explains the motion of these residues.

### Src inhibitor-1, an inhibitor of typical Src also reduces *Eh*Src activity

In silico analyses demonstrated that the kinase domain of Src is present in *Eh*Src and this sequence is a target for Src inhibitor-1. To analyze the possibility of binding between the *Eh*Src and Src inhibitor-1, docking studies were performed in MOE. This study demonstrated that Src inhibitor-1 binds to the amino acids Lys 548, Val 550, Lys 580, Glu 585, Tyr 586, Gly 608, Ile 609 and Phe 610 in the *Eh*Src (Fig. 3a). These findings indicate that Src inhibitor-1 interacts with Tyr 586 (the equivalent of Tyr 416 in a typical Src), which is responsible for the ATP binding necessary for Src activation and thereby inhibits its phosphorylation. These results suggest that Src inhibitor-1 could affect *Eh*Src activation in *E. histolytica* by binding to the catalytic domain in the site where ATP binds. To confirm this hypothesis, we used a polyclonal antibody that recognizes the phosphorylated form of Src.

Live trophozoites, with or without Src inhibitor-1 treatment, were analyzed by confocal microscopy for activated Src detection; Src inhibitor-1 reduced Src activation in live trophozoites. Furthermore, this inhibitor affected actin cytoskeleton remodeling, particularly fiber stress formation (Fig. 3b). Additionally, *Eh*Src from trophozoites, treated or not with Src inhibitor-1, was immunoprecitated with the polyclonal anti-*Eh*Src antibody and blotted with anti-phospho-tyrosine antibody. Results showed that Src-inhibitor reduced *Eh*Src phosphorylation in trophozoites, compared to DMSO-treated trophozoites (Fig. 3c). Furthermore, *Eh*Src activity was measured in the immunoprecipitate using Pro-Fluor® Src-Family kinase assays (Promega, Fitchburg, WI, USA) and results demonstrated a significant reduction (*t*-test: *t*_(4)_ = -8.174, *P* = 0.001) in *Eh*Src activity in Src inhibitor-1-treated trophozoites in comparison with DMSO-treated trophozoites (Fig. 3d). These results demonstrate that *Eh*Src can be inhibited by Src inhibitor-1, most likely by binding to the catalytic domain as it occurs in other types of Src in higher eukaryotic cells.

### Divergent *Eh*Src interacts with actin

**Fig. 4:**
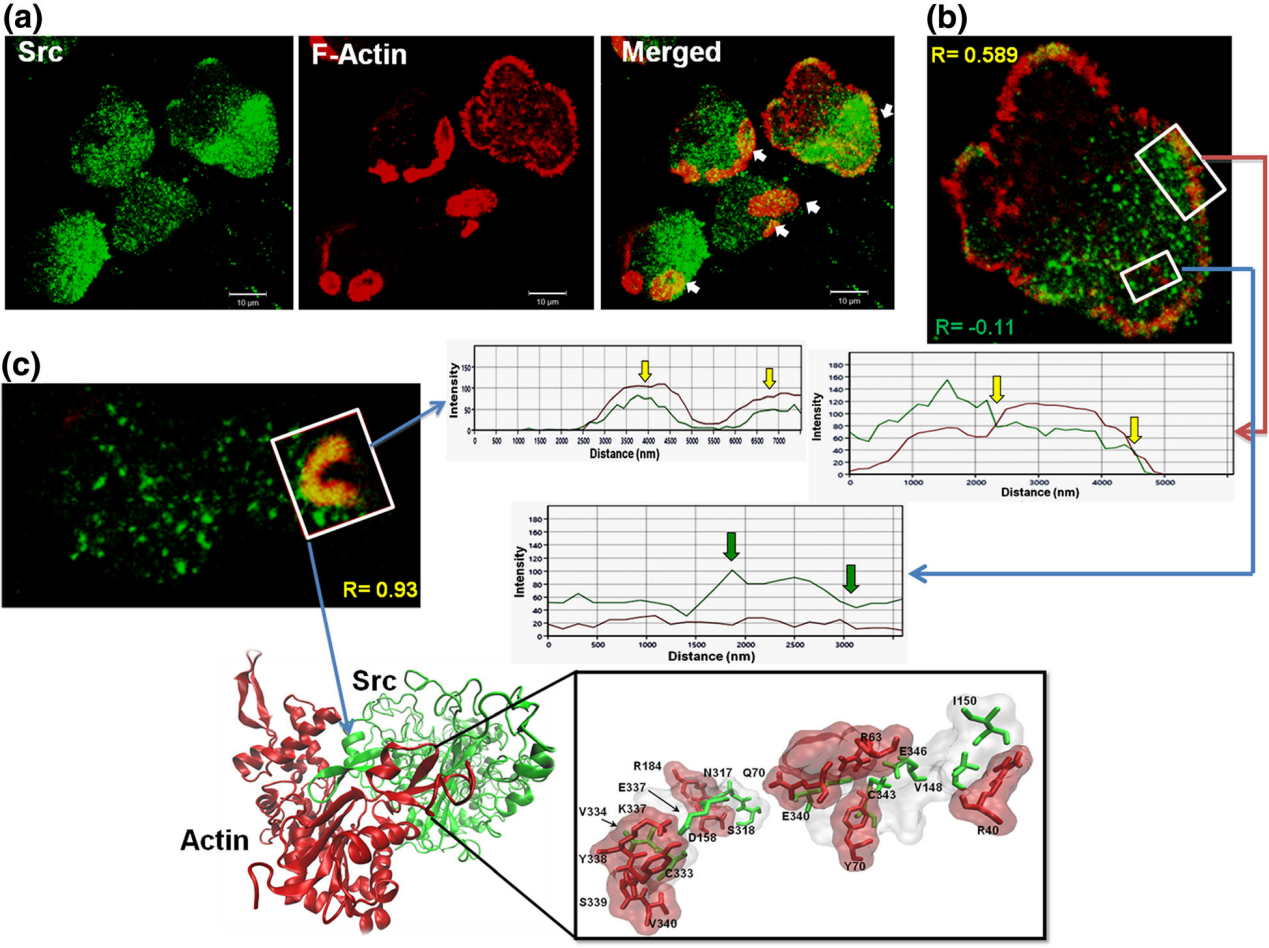
*EhSrc* interacts with actin during actin cytoskeleton reorganization. **a** Confocal microscopy analysis of trophozoites adhered to FN; actin was stained with rhodamine-phalloidin (red) (1/25), and Src (green) was detected with *Eh*Src antibody (1/250) and FITC-labeled goat anti- rabbit IgG (1/100). The white arrows in the merged image indicate the colocalization sites. At the bottom of panel (**b**) and to the right of panel (**c**), histograms of green and red intensities corresponding to selected areas (white boxes in confocal images) are shown. Areas of colocalization are marked with yellow arrows; areas where no colocalization was found are marked with green arrows. In each panel (**b** and **c**), the dispersion values indicating the results of the colocalization analyzes​​ are shown. **d** Predicted binding site of *Eh*Src and actin of *E. histolytica*. Image processing was performed with the Zen Blue software (v. 2012); the Pearson correlation coefficient was calculated with the SPSS software (v. 24). *Abbreviation*: R, Pearson correlation coefficient. *Scale-bars*: 10 μm

*Eh*Src contains KELCH domains, which typically are involved in protein-protein interactions and are also largely involved in the regulatory functions of the cytoskeleton. To corroborate the *Eh*Src kinase participation in actin cytoskeleton regulation and its binding to actin, we analyzed fibronectin adhered trophozoites using confocal microscopy to identify the presence of *Eh*Src using an anti-*Eh*Src antibody during actin cytoskeleton remodeling. Results demonstrate that *Eh*Src kinase co-localizes with F-actin during actin cytoskeleton remodeling (Fig. 4), mainly in actin structures such as cortical actin (Fig. 4b) or phagocytic invaginations (Fig. 4c), where the Pearson coefficient R values were 0.589 and 0.93, respectively. In contrast, within a representative cytoplasm region, there was no correlation between both proteins (*R* = -0.11). The docking analysis suggests the possible binding between actin and *Eh*Src through involvement of different amino acids, including amino acids of KELCH 1 domain, V148 and I150, which interact with R40 of actin (Fig. 4d; Additional file 1: Figure S1). KELCH domains are conformed by a set of five to seven KELCH repeats that form a β-propeller tertiary structure [32]. However, in this analysis it was not possible to identify the β-propeller tertiary structure characteristic of KELCH proteins, within the KELCH 1 domain of *Eh*Src kinase, at least theoretically. However, here we found that this domain participates in its interaction with actin, and some of the residues of the *Eh*Src protein which are involved in the binding to actin belong to the kinase domain (Additional file 1: Figure S1). In *Drosophila,* KELCH protein is an actin filament cross-linking protein essential for the organization of actin via Src kinase [36, 37].

Actin-*Eh*Src interaction was corroborated by co-immunoprecipitation of *Eh*Src and actin (Fig. 3c), which suggests that *Eh*Src protein could participate in the actin cytoskeleton regulation, even though it has an unusual amino acid sequence. However, the possible mechanism by which *Eh*Src regulates the actin cytoskeleton could be different from that reported in other higher eukaryotic cells, where KELCH proteins are phosphorylated by Src to regulate the actin-KELCH interaction [37].

Furthermore, Src inhibitor-1 provokes disruptions in the actin cytoskeleton remodeling during trophozoites adhesion (Fig. 3b), which we have previously reported [8], supporting our hypothesis that *Eh*Src kinase, in spite of having an unusual structure, is able to regulate the actin cytoskeleton remodeling.

### *Eh*Src participates in the formation of structures involved in erythrophagocytosis, and in encystment, but not in amoebic adhesion

**Fig. 5:**
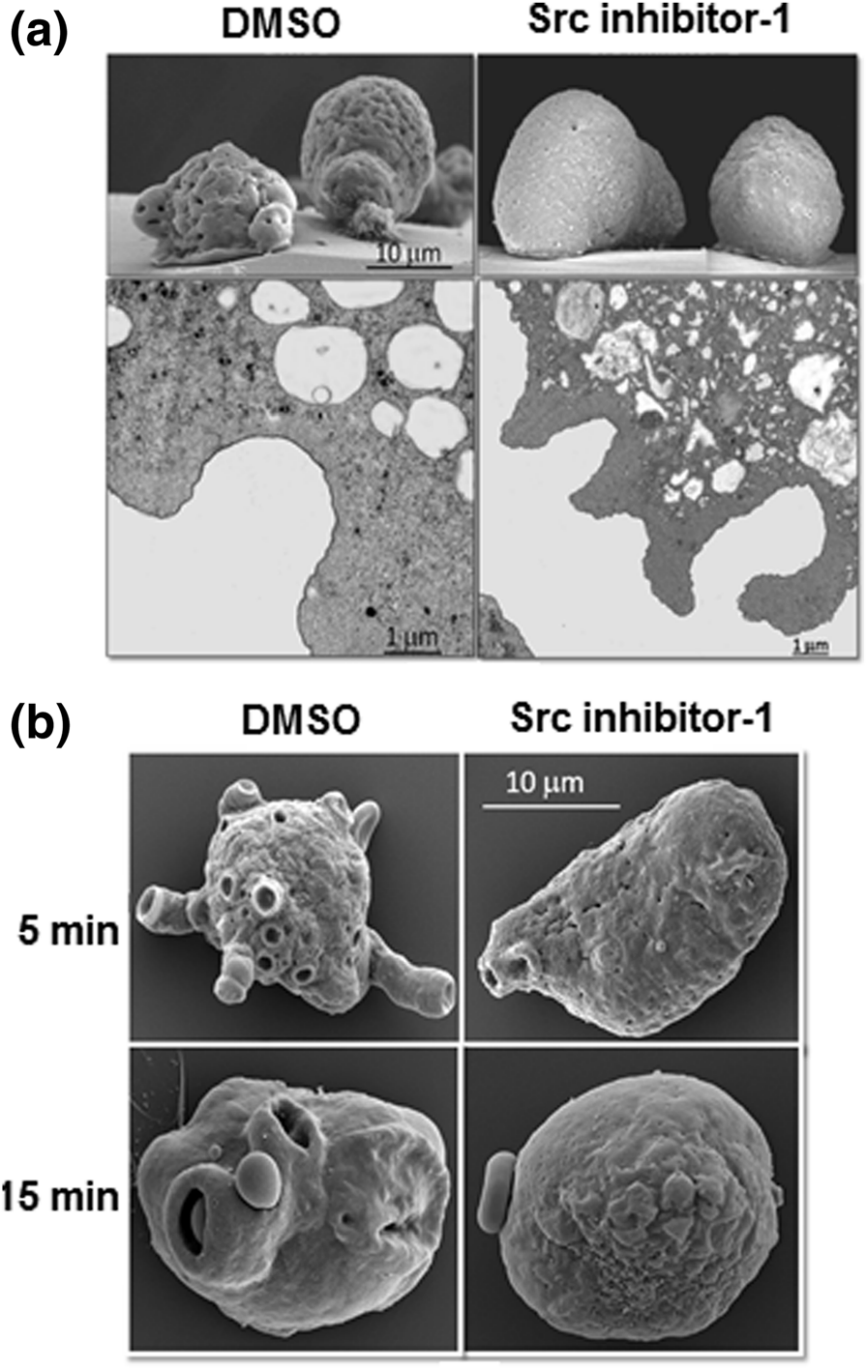
*Eh*Src participates in the formation of structures involved in erythrophagocytosis but not in amoebic adhesion. **a** Upper panel: scanning electron microscopy; lower panel: transmission electron microscopy analyses of trophozoites of *E. histolytica* with or without Src inhibitor-1 treatment adhered to nylon grids. **b** Trophozoites of *E. histolytica* with or without Src inhibitor-1 treatment interacted with human erythrocytes for 5 min (upper panel) or 15 min (lower panel), analyzed by scanning electron microscopy

Typically, Src kinases act downstream of integrin receptors and are implicated in cell adhesion [7]; thus, we evaluated the role of Src inhibitor-1 in the cell adhesion of *E. histolytica*. The results demonstrate that inhibition of *Eh*Src activity did not affect adhesion of *E. histolytica* trophozoites (Additional file 2: Figure S2). When observed by scanning electron microscopy, trophozoites treated with DMSO exhibited a diverse morphology with wrinkled cell surfaces and numerous holes (crater-like depressions); these trophozoites also developed filopodia and lamellipodia in the basal area. However, Src inhibitor-1 causes remarkable ultrastructural changes in trophozoites: the cell surface becomes smoother, the holes or crater-like depressions are significantly reduced, the filopodia are absent and the lamellipodia are shorter. Despite these differences, trophozoites treated with Src inhibitor-1 remained adhered (Fig. 5a, upper panels). Using transmission electron microscopy, we identified defects in the formation of phagocytic invaginations in Src inhibitor-1 treated trophozoites compared with DMSO-treated ones (Fig. 5a, lower panels). As observed in these images, also the number of vesicles per cell in trophozoites treated with Src inhibitor-1 diminished (Fig. 5a lower right panel) when compared with DMSO-treated trophozoites. To confirm this observation, intracytoplasmic vesicles were counted in 71 DMSO-treated amoebas and 124 Src inhibitor-1-treated amoebas, in semi-thin sections stained with Toluidine blue. No statistical difference was observed between trophozoites treated with Src inhibitor-1 and those treated only with the vehicle (data not shown).

Src kinase activity is present in phagocytic cells, and Src-deficient cells are less effective compared with wild-type cells in the mediation of phagocytosis [38]. Previously, a remarkable reduction in phagocytic capacity of *E. histolytica* was reported when trophozoites were treated with Src inhibitor-1 [8]; however, the mechanism by which Src inhibitor-1 inhibits this important amoebic function remains unknown. Here, trophozoites incubated with human erythrocytes were analyzed by scanning electron microscopy. DMSO-treated trophozoites developed numerous phagocytic invaginations, whereas Src inhibitor-1-treated trophozoites did not exhibit these structures or they were incomplete (Fig. 5b). These results indicate that Src inhibitor-1 affects the formation of phagocytic structures most likely via disruption of the actin cytoskeleton remodeling (Additional file 3: Figure S3).

**Fig. 6:**
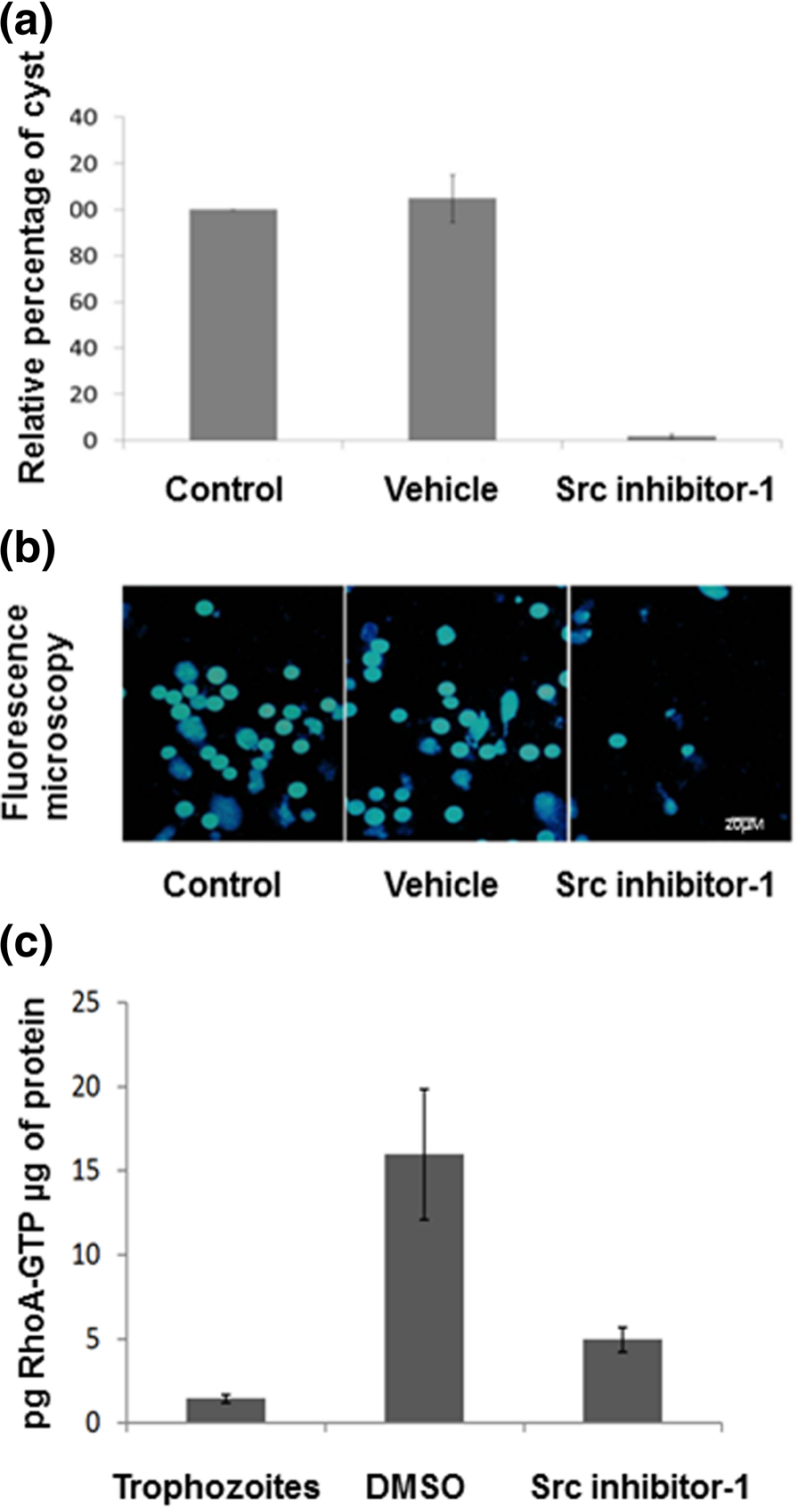
Src inhibitor-1 blocks encystment process and GTP-RhoA activity. **a** Percentage of cyst formation inhibition by Src inhibitor-1 treatment (30 μM) during 96 h. Calcofluor m2r staining of cysts produced in each condition. **b** *E. invadens* trophozoites were induced to encyst in the presence of Src inhibitor-1 or vehicle (DMSO); after 12 h of encystment, the level of RhoA-GTP was determined with a G-LISA RhoA activation assay kit (Kit no. BK121, Cytoskeleton, Inc., Denver, CO, USA), according to the manufacturer’s instructions

On the other hand, it has been demonstrated that chelerythrine chloride, staurosporine, and wortmannin inhibit the encystation of *E. invades*, which suggests an important role of kinases in this essential process of the parasite [39, 40]. We explored the potential role of *Eh*Src in the encystment of *E. invadens* (a model used to study encystation for the genus *Entamoeba*) through the use of Src inhibitor-1. Src inhibitor-1 inhibited cyst formation at 72 h post-induction of encystment, which significantly reduced the number of cysts, when compared with DMSO (Fig. 6a, b) (*t*-test: *t*_(10)_ = 23.683, *P* < 0.001).

It has been demonstrated that the actin cytoskeleton plays different roles during the encystment of *E. invadens*, such as morphological changes, transportation and disposal of vacuoles, and the transport of encystment vesicles from the cytosol to the cell wall. The actin cytoskeleton reorganization largely depends on RhoA-GTP; this result is consistent with a previous report in which the participation of RhoA-GTP was demonstrated to be important for the encystment process through actin cytoskeleton reorganization [41, 42].

The involvement of RhoA in the actin cytoskeleton organization during the early period of the encystment process of *E. invadens* has been demonstrated [41], which indicates that RhoA is essential for encystment. Therefore, we analyzed the role of *Eh*Src activity on RhoA activation during the encystment of *E. invadens*. The findings demonstrate that Src inhibitor-1 reduces RhoA activation (Fig. 6c) (*t*-test: *t*_(6)_ = 4.984, *P* = 0.002), which indicates the potential mechanism by which *Eh*Src regulates the actin cytoskeleton reorganization. Cdc42 and Rac1 activities were also assayed, but no activation could be detected during *E. invadens* encystment (data not shown).

## Conclusions

The large number of protein kinases in *Entamoeba* suggests that phosphorylation is an important regulatory mechanism of this intriguing and ancient parasite. Although *Entamoeba* has complex networks of cell signaling, it exhibits important differences compared with higher eukaryotic cells, which could be used for the design of anti-amoebic drugs to prevent transmission. While the *Eh*Src is highly divergent from its closest homologues in higher eukaryotes, it regulates important cellular functions, such as phagocytosis or the encystment process, through actin cytoskeleton regulation, which leads us to propose that amoebic kinases could be important targets to prevent amoebiasis infection.

## Additional files

Additional file 1: Figure S1. Docking analysis of *Eh*Src protein and actin. (TIFF 538 kb)

Additional file 2: Figure S2. Src inhibitor-1 did not affect adhesion of *E. histolytica* trophozoites. Trophozoites with or without Src inhibitor-1 treatment adhered to plastic during 15 min. The non-adhered trophozoites were eliminated, and the adhered trophozoites were stained with Sytox green and quantified by fluorescence. (TIFF 584 kb)

Additional file 3: Figure S3. Src inhibitor-1 inhibits phagocytic cup formation during erythrophagocytosis. *E. histolytica* trophozoites with or without Src inhibitor-1 treatment and incubated with human erythrocytes for 5 min were fixed with paraformaldehyde and stained with rhodamine-phalloidin (red) (1/25). A phagocytic invagination is clearly seen in the non-treated trophozoite (left panel) in comparison with the treated one where such structures cannot be seen (right panel). (TIFF 2135 kb)

## Abbreviations

BS: Bovine serum
BSA: Bovine serum albumin
DMSO: Dimethyl sulfoxide
*Eh*Src: Divergent *Entamoeba histoyltica* Src
*Ei*Src: Divergent *Entamoeba invadens* Src
FITC: Fluorescein IsoTioCyanate
GTPase: Enzyme that binds and hydrolyzes guanosine triphosphate
hSrc: Human Src
IgG: Immunoglobulin G
MOE: Molecular Operating Environment
NAMD: NAnoscale Molecular Dynamics
PME: Particle Mesh Ewald
Rg: Radius of gyration
RMSD: Root mean square deviation
RMSF: Root mean square fluctuation
SrcA: Non-receptor tyrosine kinase
VMD: Visual Molecular Dynamics

## References

[1] Espinosa-Cantellano M, Martínez-Palomo A (2000). Pathogenesis of intestinal amebiasis: from molecules to disease. Clin Microbiol Rev.

[2] Haque R, Huston CD, Hughes M, Houpt E. Petri, W A Jr. Amebiasis. N Engl J Med. 2003;348:1565–73. 10.1056/NEJMra022710 12700377

[3] McConnachie EW (1969). The morphology, formation and development of cysts of *Entamoeba*. Parasitology.

[4] Bakker-Grunwald T, Wöstmann C (1993). *Entamoeba histolytica* as a model for the primitive eukaryotic cell. Parasitol Today.

[5] Loftus B, Anderson I, Davies R, Alsmark UC, Samuelson J, Amedeo P (2005). The genome of the protist parasite *Entamoeba histolytica*. Nature.

[6] Anamika K, Bhattacharya A, Srinivasan N (2008). Analysis of the protein kinome of *Entamoeba histolytica*. Proteins.

[7] Frame MC, Fincham VJ, Carragher NO, Wyke JA (2002). V-Src’s hold over actin and cell adhesions. Nat Rev Mol Cell Biol.

[8] López-Contreras L, Hernández-Ramírez VI, Flores-García Y, Chávez-Munguía B, Talamás-Rohana P (2013). Src and PI3 K inhibitors affect the virulence factors of *Entamoeba histolytica*. Parasitology.

[9] Tian G, Cory M, Smith AA, Knight WB (2001). Structural determinants for potent, selective dual site inhibition of human pp60c-src by 4-anilinoquinazolines. Biochemist.

[10] Larkin MA, Blackshields G, Brown NP, Chenna R, McGettigan PA, McWilliam H, Valentin F, Wallace IM, Wilm A, Lopez R, Thompson JD, Gibson TJ, Higgins DG (2007). ClustalW and ClustalX version 2.0. Bioinformatics.

[11] Goujon M, Mc William H, Li W, Valentin F, Squizzato S, Paern J, López RA (2010). A new bioinformatics analysis tools framework at EMBL-EBI. Nuc Ac Res.

[12] Roy A, Kucukural A, Zhang Y (2010). I-TASSER: a unified platform for automated protein structure and function prediction. Nat Prot.

[13] Russell RB, Barten GJ (1992). Multiple protein sequence alignment for tertiary structure comparison: assignment of global and residue confidence level. Proteins.

[14] Frisch MJ, Trucks GW. Schlegel HB. Gaussian 03, Revision C. 02, Gaussian, Wallingford, Conn, USA, 2004.

[15] Phillips JC, Braun R, Wang W, Gumbart J, Tajkhorshid E, Villa E (2005). Scalable molecular dynamics with NAMD. J Comp Chem.

[16] AD MK, Bashford D, Bellott M, Dunbrack RL, Evanseck J, Field MJ (1998). All-atom empirical potential for molecular modeling and dynamics studies of proteins. J Phys Chem B.

[17] Batcho PF (2001). The energy operator and new scaling relations for the incompressible Navier-stokes equations. SIAM J Appl Math.

[18] Comeau SR, Gatchell DW, Vajda S, Camacho CJ (2003). ClusPro: an automated docking and discrimination method for the prediction of protein complexes. Bioinformatics.

[19] Humphrey W, Dalke A, Schulten KVMD (1996). Visual molecular dynamics. J Mol Graph.

[20] Espinoza-Fonseca LM, Ilizaliturri-Flores I, Correa-Basurto J (2012). Backbone conformational preferences of an intrinsically disordered protein in solution. Mol BioSyst.

[21] Glykos NM (2006). Sofware news and updates. Carma: a molecular dynamics analysis program. J Comp Chem.

[22] Loyola PK, Campos-Rodríguez R, Bello M, Rojas-Hernández S, Zimic M, Quiliano M (2013). Theoretical analysis of the neuraminidase epitope of the Mexican a H1N1 influenza strain, and experimental studies on its interaction with rabbit and human hosts. Immunol Res.

[23] Molecular Operating Enviroment (MOE), 2013. 08; Chemical Computing Group Inc., 1010 Sherbooke St. west, suite #910, Montreal, QC, Canada, H3A 2R7, 2014.

[24] Holtzhauer M (2006). Basic methods for the biochemical lab.

[25] Diamond LS, Harlow DR, Cunnick CCA (1978). New medium for the axenic cultivation of *Entamoeba histolytica* and other *Entamoeba*. Trans Roy Soc Trop Med Hyg.

[26] Sánchez L, Enea V, Eichinger D (1994). Identification of a developmentally regulated transcript expressed during encystation of *Entamoeba invadens*. Mol Bioch Parasitol.

[27] Flores-Robles D, Rosales C, Rosales-Encina JL, Talamás-Rohana P (2003). *Entamoeba histolytica*: a beta 1 integrin-like fibronectin receptor assembles a signaling complex similar to those of mammalian cells. Exp Parasitol.

[28] Talamás-Lara D, Talamás-Rohana P, Fragoso-Soriano RJ, Espinosa-Cantellano M, Chávez-Munguía B, González-Robles A, Martínez-Palomo A (2015). Cell-matrix interactions of *Entamoeba histolytica* and *E. dispar*. A comparative study by electron-, atomic force- and confocal microscopy. Exp Cell Res.

[29] González-Robles A, Salazar-Villatoro L, González-Lázaro M, Omaña-Molina M, Martínez-Palomo A. *Vahlkampfia* sp: structural observations of cultured trophozoites. Exp Parasitol. 2012;130(1):86–90. 10.1016/j.exppara.2011.10.009 22067209

[30] Finn RD, Coggill P, Eberhardt RY, Eddy SR, Mistry J, Mitchell AL (2016). The Pfam protein families database: towards a more sustainable future. NuclAcids Res.

[31] Montaño S, Orozco E, Correa-Basurto J, Bello M, Chávez-Munguía B, Betanzos A. Heterodimerization of the *Entamoeba histolytica* EhCPADH virulence complex through molecular dynamics and protein-protein docking. J Biomol Struct Dyn. 2017;35(3):486–503. 10.1080/07391102.2016.1151831 26861050

[32] Aguayo OR, Méndez LO, Romo MA, Castillo R, Yépez ML, Medina FJ, Hernández CA (2013). Molecular basis for benzimidazole resistance from a novel β-tubulin binding site model. J Mol Graph Model.

[33] Kumar A, Ali V, Nozaki T, Zhang KY, Bhakuni V (2012). Novel protein-protein interactions between *Entamoeba histolytica* d-phosphoglycerate dehydrogenase and phosphoserine aminotransferase. Biochimie.

[34] Bork P, Doolittle RF (1994). *Drosophila* Kelch motif is derived from a common enzyme fold. J Mol Biol.

[35] Kelso RJ, Hudson AM, Cooley L (2002). *Drosophila* Kelch regulates actin organization via Src64-dependent tyrosine phosphorylation. J Cell Biol.

[36] Hunter S, Huang MM, Indik ZK, Schreiber AD (1993). Fc gamma RIIA-mediated phagocytosis and receptor phosphorylation in cells deficient in the protein tyrosine kinase Src. Exp Hem.

[37] Makioka A, Kumagai M, Ohtomo H, Kobayashi S, Takeuchi T (2001). Inhibition of encystation of *Entamoeba invadens* by wortmannin. Parasitol Res.

[38] Makioka A, Kumagai M, Kobayashi S, Takeuchi T (2003). Involvement of signaling through protein kinase C and phosphatidylinositol 3-kinase in the excystation and metacystic development of *Entamoeba invadens*. Parasitol Res.

[39] Herrera-Martínez M, Hernández-Ramírez VI, Lagunes-Guillén AE, Chávez-Munguía B, Talamás-Rohana P (2013). Actin, RhoA, and Rab11 participation during encystment in *Entamoeba invadens*. Biomed Res Int.

[40] Garcia-Mata R, Boulter E, Burridge K (2011). The ‘invisible hand’: regulation of RHO GTPases by RHOGDIs. Nat Rev Mol Cell Biol.

