# Supplementary figures and images for: Structural and functional characterization of the divergent Entamoeba Src using Src inhibitor-1
Source: Parasit Vectors. 2017 Oct 18;10:500. doi: 10.1186/s13071-017-2461-5 (PMC5648430; doi:10.1186/s13071-017-2461-5)

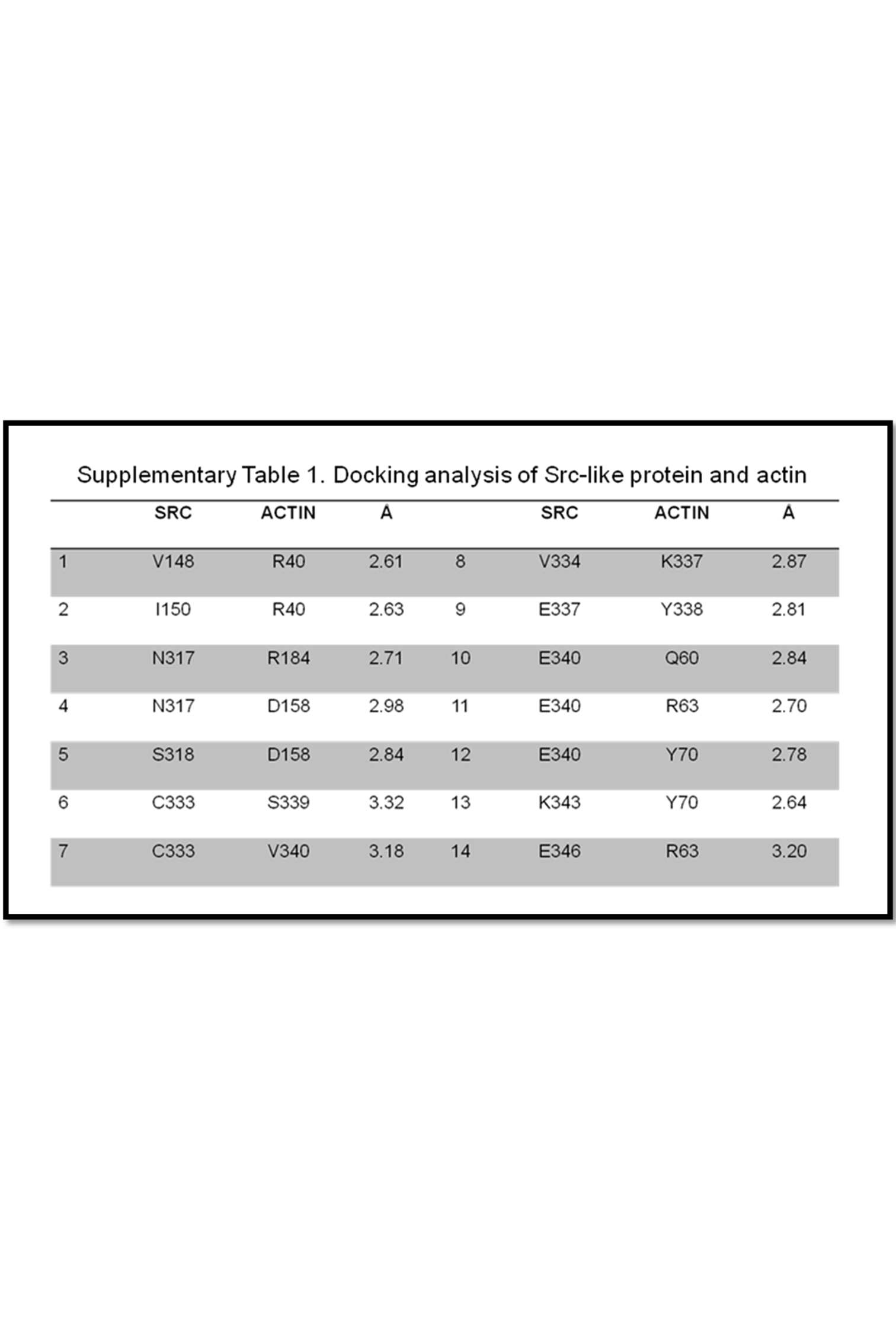

Supplement: Supplementary file 1 — Docking analysis of EhSrc protein and actin. (TIFF 538 kb) [file 13071_2017_2461_MOESM1_ESM.tif]

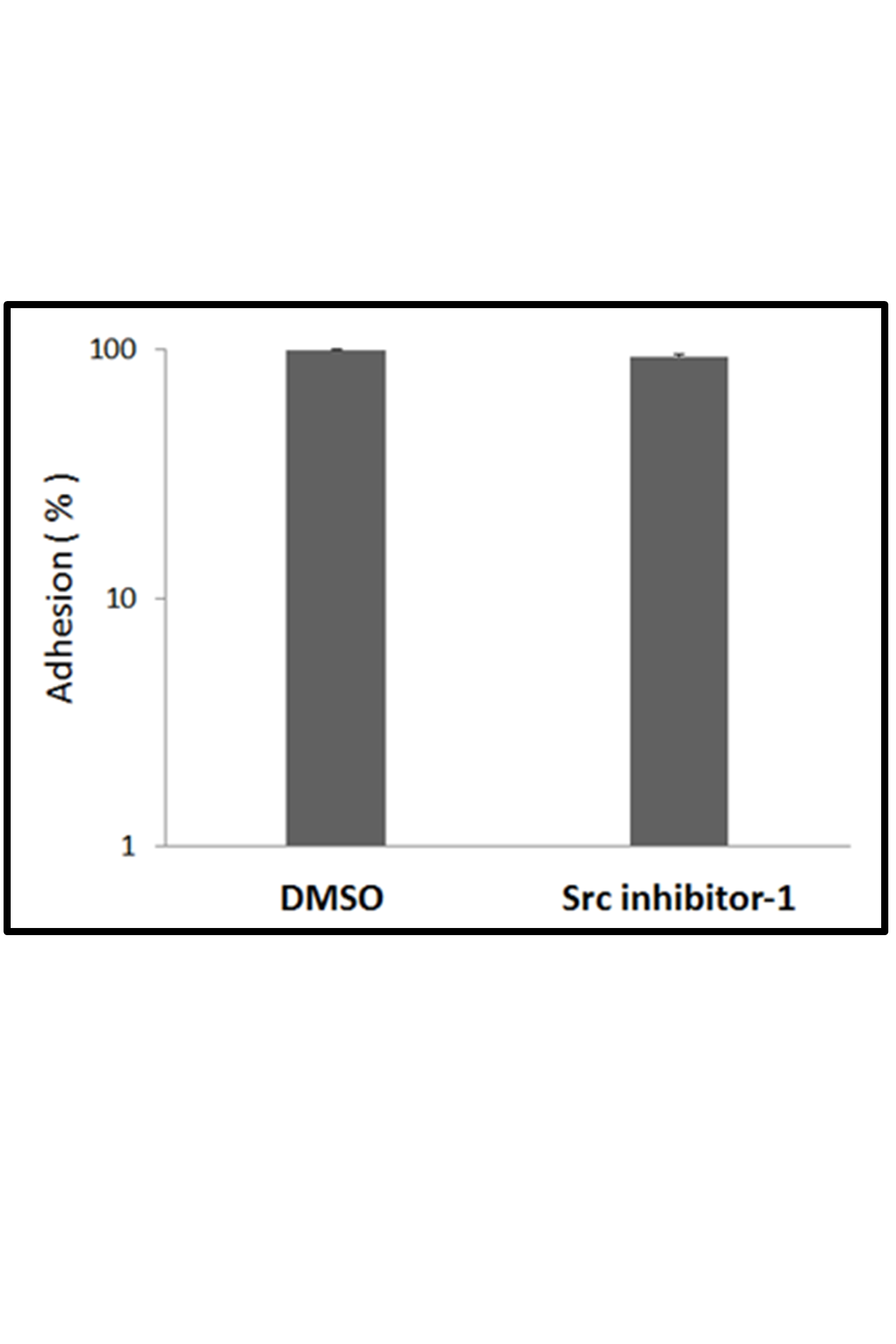

Supplement: Supplementary file 2 — Src inhibitor-1 did not affect adhesion of E. histolytica trophozoites. Trophozoites with or without Src inhibitor-1 treatment adhered to plastic during 15 min. The non-adhered trophozoites were eliminated, and the adhered trophozoites were stained with Sytox green and quantified by fluorescence. (TIFF 584 kb) [file 13071_2017_2461_MOESM2_ESM.tif]

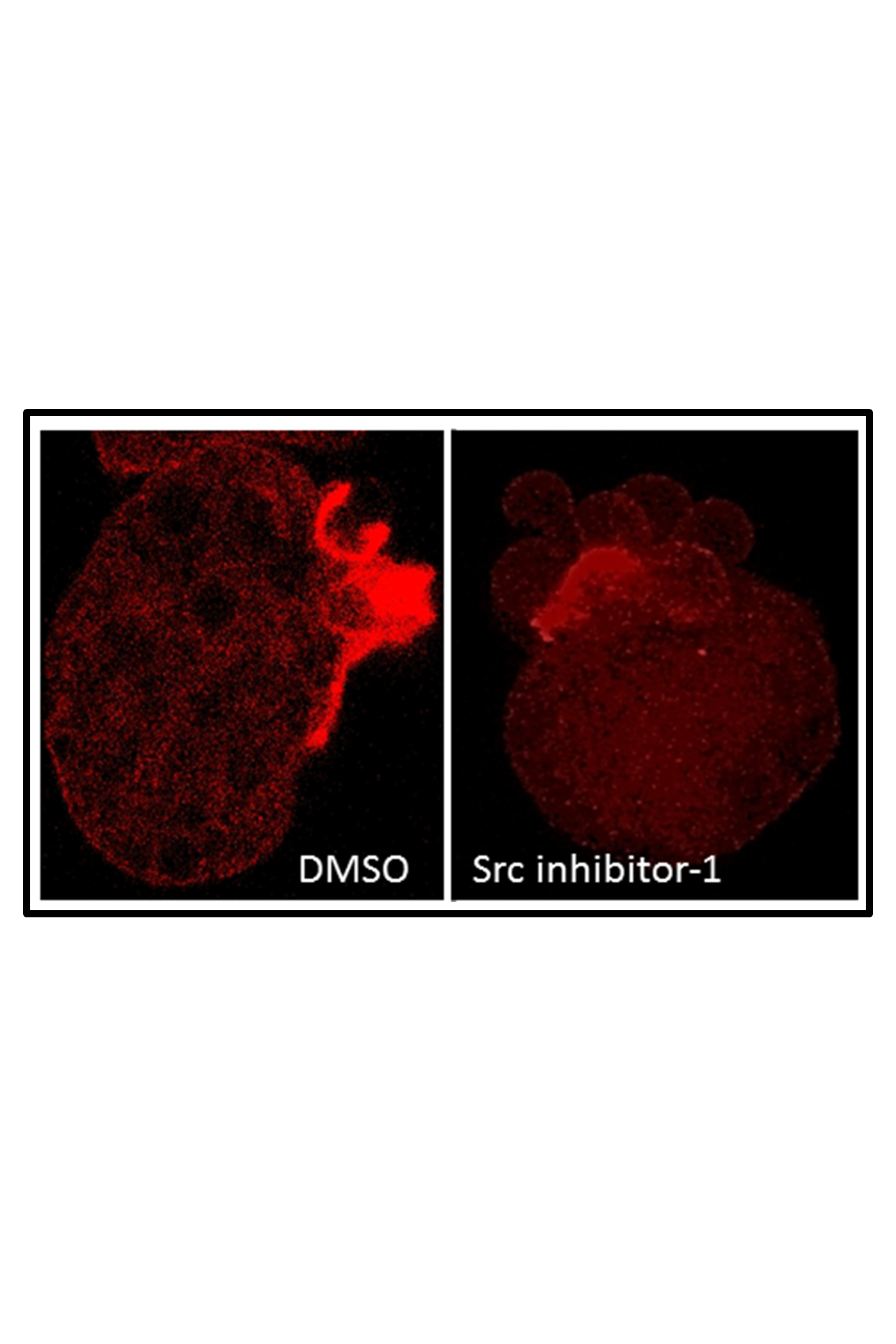

Supplement: Supplementary file 3 — Src inhibitor-1 inhibits phagocytic cup formation during erythrophagocytosis. E. histolytica trophozoites with or without Src inhibitor-1 treatment and incubated with human erythrocytes for 5 min were fixed with paraformaldehyde and stained with rhodamine-phalloidin (red) (1/25). A phagocytic invagination is clearly seen in the non-treated trophozoite (left panel) in comparison with the treated one where such structures cannot be seen (right panel). (TIFF 2135 kb) [file 13071_2017_2461_MOESM3_ESM.tif]
